# Negri Bodies in Rabies

**Published:** 1906-05

**Authors:** James N. Brawner

**Affiliations:** Atlanta, Ga., Physician in Charge Georgia Pasteur Institute


ATLANTA

### Journal-Record of Medicine

Successor to Atlanta Medical and Surgical Journal, Established 1855, and Southern Medical Record, Established 1870.

OWNED BY THE ATLANTA MEDICAL JOURNAL CO.

Published Monthly.

VOL. VIII. MAY, 1906. No. 2.

BERNARD WOLFF, M.D., EDITOR, Nos. 319-20 Prudential.

M. B. HUTCHINS, M.D., BUSINESS MANAGER, 1007-1008 Century Bldg.

E. G. BALLENGER, M.D., ASSOCIATE EDITOR AND ASSISTANT MANAGER.

### ORIGINAL COMMUNICATIONS.

INDEX THROUGH

### NEGRI BODIES IN RABIES.*

BY JAMES N. BRAWNER, M.D., ATLANTA, GA.,
Physician in Charge Georgia Pasteur Institute.

In March, 1903, Dr. A. Negri, of the University of Pavia, announced the discovery of certain bodies in the central nervous system of animals dead of rabies, and he believed them to be the specific germ of this disease. This announcement, coming from Dr. Golgi's assistant and from one perfectly familiar with the microscopical appearances of both normal and pathologic nerve tissue, at once attracted attention, and since that time numerous investigators, especially the Italians, have confirmed his results. The structures described by Negri are usually found in the cytoplasm of nerve cells or the proximal ends of their processes, but not in the nucleus, though they may be in contact with it. He believes them to be protozoa.

In August, 1903, Dr. Negri made a further report, stating, to

*Read before the Fulton County Medical Society.

that time he had examined the nervous systems of over one hundred animals suffering from rabies and various other diseases, and found the bodies only in the animals proven to be rabid by the inoculation test. In two instances where rabies existed the structures could not be demonstrated.

Later, Bertarelli and Volpino found the bodies described by Negri in the brain of a lad that succumbed to hydrophobia. Bertarelli, summarizing the work done in Italy by Negri, Volpino, Abba, Bermans and himself, states that in more than a thousand tests the bodies were found in all cases of rabies except three, and were never found in animals free from the disease as determined by the inoculation of rabies.

Poor, in New York, after twenty-three examinations, confirmed the results of the Italians.

The structures described by Negri are most frequently found as inclusions of the large pyramidal cells of the hippocampus major, Purkinje cells of the cerebellum, the large cells of the spinal ganglia, spinal cord, pons, and occasionally the pyramidal cells of the cerebral cortex. As a rule, they are much more numerous in the hippocampus major than in other portions of the nervous system, though this is not always the case, as they may be absent here and numerous in the cerebellum or spinal cord. Negri states that when rabies is produced by inoculation into the sciatica nerve, the bodies are found chiefly in the spinal cord and ganglia, rarely in the brain. When the inoculation is on the face, they are more abundant in the gasserian ganglia, hippocampus major, and cerebellum. There seems to be a difference of symptoms corresponding to the distribution of the bodies. Ordinarily, when the disease is acquired in the natural manner, they are larger and more numerous in the hippocampus major. This is probably due to the fact that most bites communicating the disease are inflicted on the head or face.

As usually found, Negri bodies are from four to six microns in diameter. Occasionally, especially early in the disease, they are very small, barely visible with a one-twelfth oil immersion. At other times they are large, measuring twenty-five microns in length. The larger forms are more frequently found in laboratory rabies and in the natural form at a point closest to the site of inoculation.

They are round or oval in shape, occasionally irregular or elongated. When seen in fresh, unstained tissue, teased in a dilute acetic acid solution, they seem to consist of non-granulated substance of hyaline appearance. In the center of the larger forms bright spots may be seen, surrounded by a band of homogeneous protoplasm. In specimens stained with eosin and methylene blue, they appear as dark red structures imbedded in the blue protoplasm of the nerve cells. They show up black in specimens stained with osmic acid.

To demonstrate these bodies, fix a small piece of each the hippocampus major and cerebellum in a saturated solution of corrosive sublimate, or of Zenkers fluid, dehydrate in absolute alcohol for six or eight hours, and section in paraffin. The sections should be thin and uniform. Fix upon a slide with Mayer's albumin, treat with a one-half per cent. aqueous solution of eosin for five minutes, wash in water and counter-stain with one-half per cent. aqueous solution of methylene blue for five minutes. Rinse in water, dehydrate in absolute alcohol, clear in xylol and mount in balsam. In specimen thus prepared, the Negri bodies stand out very distinctly as dark red structures, imbedded in the blue cytoplasm of nerve cells. As a rule, but one body is found in a cell, though occasionally two, three, or even four may be seen. The smaller bodies appear homogeneous in structure, but the larger ones contain a blue central mass resembling a nucleus.

While most observers believe these bodies to be the causative factor in rabies, all admit that positive proof is lacking. The definite morphology of the structures and the constancy of their occurrence tend to substantiate such a belief. Three hypotheses may be offered to explain their occurrence: (1) Degenerations, or artefacts of the cell protoplasm; (2) Phagocytosis, that is, the nerve cells attracting and enveloping certain other normal histological structures, e. g., red blood corpuscles; (3) The specific micro-organism of the disease.

As to the first hypothesis Negri states that artefacts can not be made by any method of preparation to simulate these bodies, and his conclusions are corroborated by all observers so far as I am able to determine. The question of degenerations should be carefully considered. While it is the general opinion that no form of metamorphosis of the cell protoplasm could account for structures so constantly found in certain cells, so definite in shape and uniform in staining reaction, yet, the possibility of the toxin causing a specific form of degeneration should be kept in mind.

Poor states that in a case of tetanus minute eosinophilic granules were found in the Perkinje cells of the cerebellum, but to an experienced eye they would not be mistaken for Negri bodies. Nerve cells in all stages of degeneration show no such structures as are found in rabies. Negri bodies retain their characteristics in cells in all stages of degeneration and Negri states that they even resist the putrefactive process.

The theory of phagocytosis is less tenable than that of specific degeneration. To the experienced, erythrocytes are most likely to be mistaken for Negri bodies, than any other histological structure, as they are about the same size, though usually larger, and are eosinophilic in reaction. This fact suggested the hypothesis that Negri bodies were red-blood corpuscles phagocytized by nerve cells. To me this view is untenable for many reasons. While both Negri bodies and erythrocytes stain red, the former are of a darker color. In fresh preparations the bodies are hyaline and have not the slightest resemblance to red-blood cells, and in sections stained with osmic acid they show up black, while the latter remain unstained. It is also impossible to conceive of a phagocytized red-blood corpuscle increasing in size and developing a nucleus. Phagocytized red-blood corpuscles in the spleen show no resemblance to Negri bodies, but are found as fragments and in various stages of degeneration.

Both direct and indirect evidence in favor of these structures being the specific germs of the disease may be said to exist. As stated by Bertarelli, "In more than a thousand tests the Negri bodies were never found in animals free from rabies as determined by inoculation of rabbits. On the other hand they were always found in infected animals with three exceptions, and in these only a part of the nervous system had been examined." It may be said that in cases of rabies if all parts of the nervous system were examined, these bodies would invariably be found. All observers agree that Negri bodies are never found in other diseases, even in those where the degenerative changes in the nerve cells are pronounced. Judging from analogy we must admit that rabies is caused by a specific micro-organism; and from its behavior to heat, chemicals and culture media, we are probably justified in believing it to be a protozoan.

The virus is destroyed in a few minutes at a temperature of 110° F. Two per cent. carbolic acid renders it inert in a few seconds, and it is rapidly destroyed by any of the weaker antiseptics. It retains its virulence for weeks or months in neutral glycerine. Glycerine that is slightly acid destroys the virus. It can not be grown on any known culture medium.

Like many protozoan diseases, it is communicated through the saliva. We now know that malaria, mountain fever, cattle fever, and probably yellow fever, are protozoan diseases, and are all communicated through the saliva of infected mosquitoes, ticks, etc. While it is true that the virus of rabies does not pass through an intermediate host, but directly from animal to animal, yet the fact that it is eliminated by way of the salivary glands is suggestive of the protozoan nature of the organism. So, from indirect evidence in our possession, the specific germ of rabies is probably a protozoan, and as Negri bodies resemble protozoa, we are justified in assuming them to be the causative factor in this disease.

Whether or not we regard the bodies discovered by Negri as the specific cause of rabies, their value as a means of a rapid diagnosis is now generally recognized. When the brain of an animal is examined and Negri bodies are found, the evidence is considered positive and a diagnosis of rabies is made. More than one thousand tests confirmed by the inoculation of rabbits have now been reported, and not a single exception has been found. I have demonstrated Negri bodies in thirty-four dogs and two cats, and in every instance the rabbits inoculated die of typical rabies. In three cases where the inoculation test resulted positively, no bodies were found, though only the hippocampus major was examined and the animals were killed in the first stages of the disease. In over a dozzen dogs and cats proven not to be rabid, no such structures could be demonstrated.

DISCUSSION OF DR. BRAWNER'S PAPER.

*Dr. Claude Smith:* I have listened with a great deal of pleasure to the paper by Dr. Brawner. The discovery of these Negri "bodies" is of considerable interest, as it seems to furnish a means of early diagnosis of rabies in animals. In the past we have had to rely upon the inoculation of animals for a positive diagnosis, and as this takes three or four weeks to reach a conclusion, you can readily see that a means for making an early diagnosis was much desired and greatly needed. On account of the variety of symptoms which the disease produces, and also because many other conditions may produce almost the same symptoms, it is often impossible to say whether a suspected animal was suffering with rabies or not.

In view of this great need of a means of early diagnosis of the disease, various investigators have endeavored to discover some means whereby this might be accomplished. Babes reported finding the so-called "tubercles," consisting of a perivascular small cell infiltration which he claimed to be diagnostic of the disease. His views were supported by many good men, but the method has never proven satisfactory.

Nélis and Van Gehutchten reported finding the degeneration of the ganglia cells with a proliferation of the endothelial cells lining the capsules, and which they considered as diagnostic of the disease in connection with the symptoms of the suspected animal. This method was also supported by many, but it did not meet with universal approval, as it was found that other diseases might produce the same changes.

In the reports upon these Negri bodies all investigators report that the presence of these bodies, especially in the hippocampus major is constant whenever the disease is present, as proven by the inoculation of animals, and that they are found only in this disease.

Whether these bodies are the parasite of the disease is a disputed question among the various investigators, some claiming that as the "virus" of rabies will pass through a Pasteur-Chamberlin filter, that therefore these bodies are too large to be the infective agent.

However, as before stated, they all agree as to the constancy of these "bodies" in the disease, and as to their diagnostic value. From these reports it appears that we here have as sure a means of diagnosing rabies by the presence of the Negri "bodies" as we have of diagnosing syphilis by the presence of the spirocheta pollida.

Therefore, instead of being compelled to wait in anxious suspense for three or four weeks, the patient can now be advised within a few hours whether the suspected animal was suffering with rabies or not.

*Dr. Brawner* (closing) : I wish to confirm the statements made by Dr. Smith in reference to the "rabic tubercle" described by Babes, and the cellular proliferation about the ganglion cells, first noticed by Van Gehutchten and Nélis. These findings are not characteristic of rabies and they are frequently hard to demonstrate, and are practically of no value in the diagnosis of their disease. It is difficult, however, with Negri bodies, as they have been found in no other disease, are easy to find, and are now regarded as diagnostic of rabies.

---

THE greatest immediate danger after a tracheotomy is the possibility of a subsequent pneumonia. This can, in a large measure, be obviated by filtering the inspired air through a soft sponge saturated with warm one per cent. phenol solution.—*American Journal of Surgery.*

